# Who pays for and who benefits from health care services in Uganda?

**DOI:** 10.1186/s12913-015-0683-9

**Published:** 2015-02-01

**Authors:** Brendan Kwesiga, John E Ataguba, Christabel Abewe, Paul Kizza, Charlotte M Zikusooka

**Affiliations:** HealthNet Consult, P.O. Box 35928, Kampala, Uganda; Health Economics Unit, School of Public Health and Family Medicine, University of Cape Town, Cape Town, Observatory 7925 South Africa

**Keywords:** Universal coverage, Equity in health financing, Benefit incidence analysis, Uganda

## Abstract

**Background:**

Equity in health care entails payment for health services according to the capacity to pay and the receipt of benefits according to need. In Uganda, as in many African countries, although equity is extolled in government policy documents, not much is known about who pays for, and who benefits from, health services. This paper assesses both equity in the financing and distribution of health care benefits in Uganda.

**Methods:**

Data are drawn from the most recent nationally representative Uganda National Household Survey 2009/10. Equity in health financing is assessed considering the main domestic health financing sources (i.e., taxes and direct out-of-pocket payments). This is achieved using bar charts and standard concentration and Kakwani indices. Benefit incidence analysis is used to assess the distribution of health services for both public and non-public providers across socio-economic groups and the need for care. Need is assessed using limitations in functional ability while socioeconomic groups are created using per adult equivalent consumption expenditure.

**Results:**

Overall, health financing in Uganda is marginally progressive; the rich pay more as a proportion of their income than the poor. The various taxes are more progressive than out-of-pocket payments (e.g., the Kakwani index of personal income tax is 0.195 compared with 0.064 for out-of-pocket payments). However, taxes are a much smaller proportion of total health sector financing compared with out-of-pocket payments. The distribution of total health sector services benefitsis pro-rich. The richest quintile receives 19.2% of total benefits compared to the 17.9% received by the poorest quintile. The rich also receive a much higher share of benefits relative to their need. Benefits from public health units are pro-poor while hospital based care, in both public and non-public sectors are pro-rich.

**Conclusion:**

There is a renewed interest in ensuring equity in the financing and use of health services. Based on the results in this paper, it would seem that in order to safeguard such equity, there is a need for policy that focuses on addressing the health needs of the poor while continuing to ensure that the burden of financing health services does not rest disproportionately on the poor.

## Background

Universal coverage for health care has been emphasised as a major policy goal for all health systems. Recently, this resolve has been expressed in the United Nations General Assembly resolution 67/123 on universal coverage [1]. Universal coverage entails *inter alia*, guaranteed access to needed health care that is of acceptable quality for all while eliminating or significantly limiting exposure to financial risk [2]. This requires that payment for health care should be according to the capacity to pay while utilisation is according to need [3]. There is therefore a necessity for income cross-subsidisation from the rich to the poor and risk-related cross-subsidisation from the healthy to the sick. Such cross-subsidisation occurs in health systems mainly financed through prepayment mechanisms [2]. However, in most developing countries including Uganda, prepayment arrangements are scarce. Health care services are therefore paid formainly through direct out-of-pocket payments.

In Uganda, health service is delivered through both the public and private sectors. As in many other African countries, the role and prominence of the private sector is increasing. This is attributed *inter alia* to both the perceived and the existing inefficiencies in the public sector [4-6]. Private facilities (comprising private for profit (PFP) and private not for profit providers/non-government organisations (PNFP/NGOs)) are utilised by both the rich and the poor [7,8]. Health care services are financed through a combination of direct out-of-pocket payments, general tax revenue and donor funding. Out-of-pocket payments as a proportion of total domestic health expenditure have been high and are increasing [9]. This appears to be a paradox given that user fees for health services in public facilities were abolished in 2001 [10]. On the other hand, the contributions from general government taxes are low with a generally decreasing trend [9]. While Uganda is a signatory to the Abuja protocol requiring African governments to allocate 15% of their budgets to the health sector, this target has not been met. It has generally accounted for approximately 9% of the country’s budget. This is even lower than is considered more realistic in budgetary allocation targets set in the country’s health sector strategic plans [11]. Prepayment arrangements in the form of voluntary community based health insurance schemes and private health insurance are generally insignificant [12]. For example, in 2009 prepayment schemes accounted for about 0.2% of total health expenditure [9]. The latest statistics indicate that only 2% of the population is insured [13].

While a few studies have examined the distribution of public subsidy in Uganda [14,15], there is a dearth of studies interrogating the distribution of both public and private subsidies. Furthermore, little is known about the distribution of the health financing burden between socio-economic groups in Uganda. Only a limited number of studies have examined the distribution of taxes in general [16,17]. Within the context of universal coverage, and the need to ensure that both sectors (private and public) work towards attaining equity in the delivery and financing of health services, this study jointly assesses equity in financing and in the distribution of health care benefits in Uganda. This is accomplished by considering all the main domestic sources of health financing and including both the public and private sector health providers.

## Methods

### Data sources

The main data source for this study is the nationally representative Uganda National Household Survey 2009/10 (UNHS IV) conducted by the Uganda Bureau of Statistics (UBOS) between May 2009 and April 2010. This survey collects comprehensive data on households including the consumption expenditure and health seeking behaviour of members. The UNHS IV used a two-stage sampling design. In the first stage 712 enumeration areas are selected using probability proportional to size based on Uganda’s 2002 national census. In the second stage, 10 households (i.e., the ultimate population sampling unit) are selected from each enumeration area by systematic random sampling. The UNHS IV covers a sample of 6800 households. The UNHS IVdata are publically available on the UBOS website (http://www.ubos.org/unda/index.php/catalog/51).

### Measurement of socio-economic status

There is debate around the suitability of the different measures of socio-economic status [18] for health equity analysis. However, consistent with similar studies on the subject under study [19,20], and in the context of a developing country, this study uses monthly per adult equivalent consumption expenditure. An alternative popularly used measure of socio-economic status is a composite socio-economic index. However, this index cannot be used to compute progressivity indices [18].

The equivalence scale used to construct the adult equivalent consumption expenditure in this paper is similar to that used by Appleton and colleagues [21]. Household consumption expenditure is adjusted based on age as represented by the relative calorie requirements^a^. The equivalence scale used is estimated as:

1 $$ EQ=\left(A+\gamma C\right) $$

where *A* represents the number of household members aged 18 years and above while *C* represents those below 18. The relative weight accorded to children, $\gamma$, varies from 0.273 for the household members below 1 year to 0.95 for household members between 16 and 18 years. In this paper no adjustment for economies of scale is made (i.e., full economies of scale is assumed). This is in line with Deaton and Zaidi [22] that the economies of scale parameter approaches unity for households in developing countries.

### Financing incidence analysis

Financing incidence analysis is concerned with which socio-economic group bears the burden of health financing in terms of contributions through taxes, direct out-of-pocket payments and insurance. Taxes considered in this study include direct taxes (personal income tax and corporate tax) and the indirect taxes (value added tax (VAT) and excise taxes). Health insurance, as noted earlier, is not considered because its contribution to health financing in Uganda is insignificant.

In this study, in line with previous studies [19,20,23], it is assumed that the burden of direct taxes falls on the parties legally targeted by the taxes. Personal income tax is computed from reported gross income based on the assumption that tax is charged on the incomes of all those identified as employed in the formal sector who are also above the taxable threshold. Reported income tax payment is not used in this study because it is not reliable in low-income countries due to under reporting of tax payments [24]. Corporate tax is computed based on the reported dividends received by household members [24]. Indirect taxes on the other hand are calculated using their respective tax rates and based on household consumption of goods on which these taxes are levied. Following [24], all estimated taxes are adjusted to reflect that reported by Uganda’s Ministry of Finance, Planning and Economic Development based on the proportionate share of the contribution of the tax components to total tax. Furthermore, we apply the proportion of public health expenditure (as indicated by the budget allocation to health) to the total tax payments so as to obtain an estimate of taxes apportioned to health. However, it is important to note that such an adjustment does not affect the distributive indices computed in this study.

The incidence of direct out-of-pocket payments is estimated based on reported household out-of-pocket payments. The progressivity of health financing is assessed using the Kakwani index [25]. This index is obtained by subtracting the Gini-coefficient of household per adult equivalent consumption expenditure from the concentration coefficient of each health financing mechanism [25]. The index ranges between -2 and 1. A positive Kakwani index indicates that a health financing mechanism is progressive while a negative Kakwani index indicates a financing mechanism which is regressive. A zero index indicates proportionality [18]. An important property of the Kakwani index is that it is additively separable. Thus, by computing the Kakwani index for each health financing mechanism, weighting it for the contribution of that financing mechanism in the total and summing them up, one can obtain the overall progressivity of the health system. The Kakwani index for the health financing sources (*π*) is computed using the convenient regression approach [26].

2 $$ 2{\sigma}_r^2\left(\frac{z_i}{{\widehat{\mu}}_z}-\frac{x_i}{{\widehat{\mu}}_x}\right)=\alpha +\pi {r}_i+{u}_i $$

where *z*_*i*_ is the health care payment (e.g., out-of-pocket payment) of household *i*, *x*_*i*_ is the per adult equivalent consumption expenditure of household *i* and ${\widehat{\mu}}_z$ and ${\widehat{\mu}}_x$ are their respective estimated averages. *r*_*i*_ is the weighted fractional rank of households, ${\sigma}_r^2$ is the variance of the fractional rank.

The weighted fractional rank is computed as ${r}_i={\displaystyle \sum_{j=0}^{i-1}}{w}_j+0.5{w}_i$; where *w*_0_ = 0 and *w*_*i*_ is the relative sample weight (i.e., scaled to sum up to 1) and observations are sorted in ascending order of per adult equivalent consumption expenditure.

Dominance tests are carried out using the multiple comparison approach [27] so as to ascertain progressivity for the different health financing mechanisms.

### Benefit incidence analysis

Benefit incidence analysis assesses the distribution of health care benefits. The standard methodology described in [28] is utilised. Benefits are obtained by multiplying health care service use and unit cost of the specific service [18]. In this study, data on utilisation of health service are obtained from the UNHS IV. However, the UNHS IV merely records utilisation contingent upon reporting an illness but excludes the recording of the use of preventive services. It also does not distinguish between inpatient and outpatient care. The proportion of inpatient and outpatient visits per facility type as reported in the Uganda Demographic and Health Survey (UDHS) 2005/06 [13] is thus used to split the recorded utilisation^b^. Unit costs for public and NGO/PNFP providers are obtained from a costing study by the Ministry of Health [29]. In line with previous studies [19,20,30], we relied on reported out-of-pocket payments in the UNHS 2009/10 survey for the use of the services from pharmacies and drug shops^c^.

To assess equity in the distribution of health care benefits, the relative share of health care benefits for each socio-economic group is obtained. Concentration indices (*β*), based on the convenient regression approach [26] are generated to assess the distribution of benefits.

3 $$ 2{\sigma}_r^2\left(\frac{y_i}{\mu}\right)=\alpha +\beta {r}_i+{\varepsilon}_i $$

where *y*_*i*_ is the value of the benefit variable for individual *i*, *μ* is its estimated average, *r*_*i*_ and ${\sigma}_r^2$ remain as previously defined.

Multiple comparison approach is again used to test for statistical dominance. Distribution of health care is said to be pro-rich if health care benefits usage is mainly among the rich (positive concentration index) and is pro-poor if the usage ismainly among the poor (negative concentration index).

Because equity in health service delivery is often defined according to individual need for health care, the benefits of each socio-economic group are compared with their need for health care. In this study, the need for healthcare is assessed based on section 6 (disability, malaria and fever module) of the UNHS IV. This section captures the ability of household members to perform activities such as seeing, hearing, mobility, learning, communication, social activities and managing personal care. The information is then used to represent the individual’s self-assessed health. Reported illness is not used as a measure of need in this study due to differences in perception of illness that may arise between different socio-economic groups [31]. In order to compare benefits and need, the benefits of each quintile of socioeconomic status and the total need of each quintile are summed up and graphs constructed to enable comparison. All analyses are performed in Stata® version 12.

This study obtained ethical clearance from the Uganda National Council of Science and Technology (REF: SS 2463).

## Results

### Distribution of health financing burden

**Figure 1:**
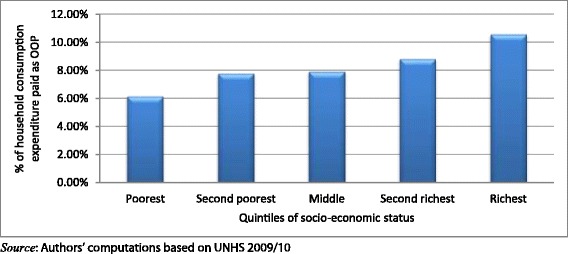
**Distribution of the burden of out**-**of**-**pocket payments across quintiles of socio**-**economic status.**

**Figure 2:**
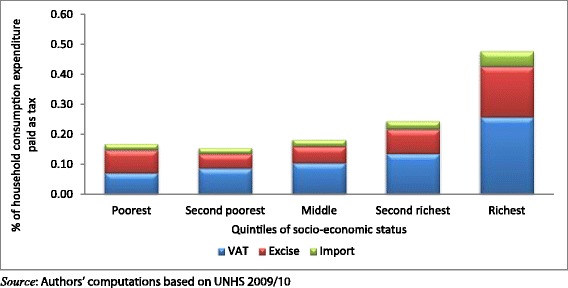
**Distribution of the burden indirect taxes across quintiles of socio**-**economic status.**

**Figure 3:**
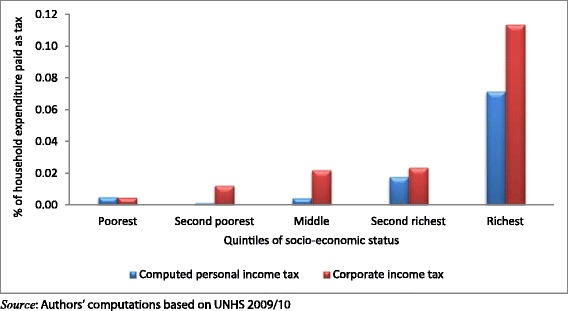
**Distribution of the burden direct taxes across quintiles of socio**-**economic status.**

The results presented in Figures 1 and 2 indicate health care payments as a proportion of households’ consumption expenditure. As shown in Figure 1, richer households spend more as a proportion of their expenditure out-of-pocket than poorer households. This distribution is similar for indirect taxes and the direct taxes as shown in Figures 2 and 3 respectively.

Using formal indices, all the different health care payment mechanisms are progressive as shown by the positive Kakwani indices and the health payments concentration curves, which dominate the Lorenz curve (Table 1). However, these payment mechanisms have varying levels of progressivity. Out-of-pocket payments are the least progressive (Kakwani index = 0.064 (p-value <0.1). Among the taxes, direct tax components (personal income tax and corporate income tax) are shown to be the most progressive. For the indirect taxes, VAT is the least progressive (Kakwani index = 0.129 (p-value <0.01)) while excise taxes are the most progressive (Kakwani index = 0.211(p-value < 0.01)).

**Table 1:** **Distribution of health financing burden**

	**% share in total**	**Concentration index** **(standard error)**	**Kakwani index** **(standard error)**	**Dominance test**	
				**45 degree line**	**Lorenz**
Out-of-pocket	73.7	0.487*** (0.023)	0.064* (0.038)	-	-
Corporate income tax	3.1	0.679*** (0.075)	0.256 (0.228)	-	-
Computed PIT	5.4	0.619*** (0.024)	0.195*** (0.068)	-	-
Excise tax	6.5	0.634*** (0.020)	0.211*** (0.029)	-	-
Import tax	2.2	0.564*** (0.019)	0.141*** (0.031)	-	-
Value added tax	9.1	0.552*** (0.014)	0.129*** (0.016)	-	-
Total	100	-	0.094	-	-

*Source*: Authors’ computations based on UNHS 2009/10.

***p < 0.01; *p < 0.1.

*Note*: - means that the 45 degree line or Lorenz curve dominates.

Although excise taxes are the most progressive of indirect taxes, some components which are not presented in this paper are regressive. Fuel, airtime and alcohol taxes are progressive while tobacco and kerosene taxes are regressive. This indicates that the burden of fuel, airtime and alcohol taxes is mainly borne by the richest households while the reverse is the case for tobacco and kerosene taxes.

Overall, as demonstrated in Table 1, Uganda’s health system is mildly progressive with Kakwani index estimated at 0.094. Although all taxes are more progressive than out-of-pocket payments, they constitute a lower financing share in total domestic financing. The overall progressivity of Uganda’s health system is thus dependent, to a larger extent, on the progressivity of out-of-pocket payments since it contributes more than 70% of domestic health financing.

### Distribution of health care benefits

**Figure 4:**
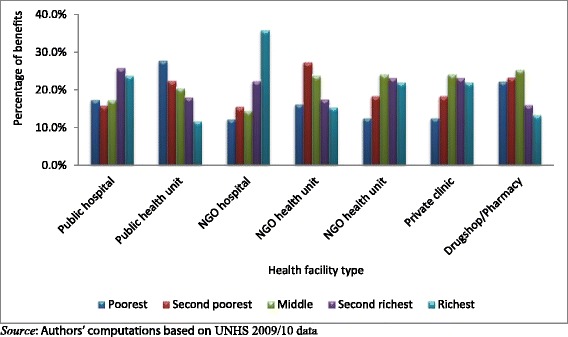
**Distribution of health benefits for each quintile of socio-** **economic status from different health care providers.**

As shown in Figure 4, the pattern of distribution of benefits varies across the different health service providers. For the public providers, the distribution of benefits from public hospitals is pro-rich with the richest quintile getting 23.7% of all benefits compared to 17.4% for the poorest. On the other hand, government health units are pro-poor with the poorest quintile getting 27.7% of all benefits as compared to 11.6% going to the richest.

For the NGO providers, the pattern is similar to that of the public providers although the distribution of NGO hospital benefits is considerably more pro-rich than that of public hospitals. For the NGO hospitals, the richest quintile gained 35.8% of all benefits as compared to 12.1% for the poorest quintile. The distribution of benefits for NGO health units is pro-poor. However, the share of benefits going to the poorest quintile is similar to that for the richest quintile.

Similarly, the distribution pattern of private clinics as shown in Figure 4 is pro-rich with the poorest quintile obtaining the least share of benefits (12.4%). Self-treatment through purchasing drugs from drug shops and pharmacies is mainly among the poor.

The pattern of distribution of health services benefits is also confirmed in Table 2 using concentration indices and dominance tests. Public health units are the most pro-poor with a concentration index of -0.167(p-value < 0.01). The use of drug shops and pharmacies is similarly pro-poor. While the NGO health units also have a negative concentration index, no clear pro-poor pattern is observed because there is non-dominance between the concentration curve and the line of equality. On the other hand, benefits from NGO hospitals are the most pro-rich with a concentration index of 0.233 (p-value < 0.01).

**Table 2:** **Benefit incidence concentration incidences and dominance results** (**All providers**)

**Provider**	**Concentration index**	**Standard error**	**Dominance test**
Public hospital	0.095**	0.075	-
Public health unit	−0.167***	0.042	+
NGO hospital	0.233***	0.116	-
NGO health unit	−0.039	0.041	Non-dominance
Private clinics	0.105***	0.053	-
Drug shops/Pharmacies	−0.091**	0.037	+

Source: Authors computations based on UNHS 2009/10.

***p < 0.01; **p < 0.05.

*Note*: - means that the 45 degree line dominates; + means that the concentration curve dominates.

**Figure 5:**
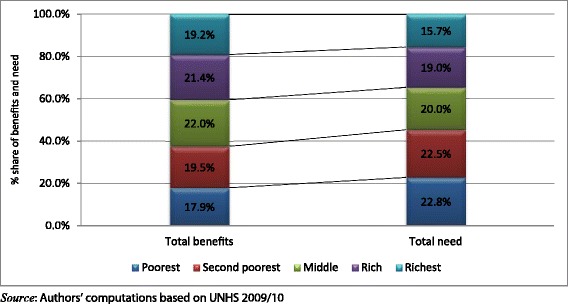
**Distribution of total benefits versus need.**

Benefits and need across the different socio-economic groups are compared and presented in Figure 5. There is an inequitable distribution of health care benefits in Uganda. The poorest quintile which needs the most health care (22.8%) has the least share of benefits (17.9%). This mismatch between need and benefit is also observed in the second poorest quintile. The remaining quintiles receive more benefit than they need and the largest difference is observed in the richest quintile.

## Discussion

Uganda’s health financing system is marginally progressive. The extent of progressivity is heavily influenced by the progressivity of out-of-pocket health payments. Out-of-pocket payments account for over 70% of total domestic health financing. This is the least progressive financing mechanism. The direct taxes are more progressive than the indirect taxes. This is because they are mainly incurred by the richer households who either earn taxable income through employment or are owners of capital (shareholders). While Uganda exempts most of the items in the consumption basket of the poor, VAT is found to be the least progressive of the indirect taxes. This is because VAT still captures other commodities consumed by these poor households. Whereas excise tax is the most progressive of the indirect taxes, its progressivity is mainly due to the progressivity of fuel, alcohol and expenditure on phone airtime. These commodities and services are mainly consumed by the rich.

The findings for both the out-of-pocket payments and taxes are in line with previous studies in low-income countries with similar structures of health financing. In Nigeria where out-of-pocket expenditure is the most dominant source of health expenditure, it was found to be progressive [32]. However, out-of-pocket payments have been found to be regressive in other African countries [19,20,23]. Similarly, in Asian countries where there are no user fees for public health providers, out-of-pocket payments were found to be progressive [18]. The implication of the progressivity of out-of-pocket payments needs to be clearly understood based on the health system under consideration. In a health system where out-of-pocket payment is the dominant payment mechanism, the rich are both more likely and more able to pay for health care than are the poor. Previous research has also demonstrated that the impact of the elimination of user fees in public facilities in Uganda was highest among the poorest indicating that user fee abolition may have protected some poor households from direct out-of-pocket payments [33]. Such progressivity however may also reflect access barriers arising from the affordability of these services for the poor.

The progressivity of taxes in Uganda is similar to those of other African countries including Ghana, Tanzania and South Africa [19,20,23]. The progressivity of these taxes implies that increasing general tax funding to the health sector will increase progressivity of the health system and greatly enhance equity in the financing of health care in Uganda.

The benefit incidence analysis results indicate a pro-rich distribution for all hospitals (public and NGOs) as well as for the private clinics. On the other hand, lower level health units particularly the public health units are pro-poor. A comparison between total benefits and need for health care indicates a maldistribution. Whereas the poor experience the greatest need, they receive the least share of benefits. The rich with the least need get the most share of benefits. Such findings are consistent with previous studies in Uganda [15] and in other African countries [3,14]. Recent studies in Tanzania, Kenya, South Africa and Ghana that considered both the public and private sectors have also indicated a pro-poor distribution of benefits for lower level public health units but a pro-rich distribution for higher level hospitals and private providers [19,23,29,34].

The difference in the distribution of the health sector benefits may be explained by various factors. The formal health sector providers who are geographically more accessible to the population and provide free services (charge no user fees) are found to be pro-poor. This points to availability and affordability of health care as key determinants of access to health care in Uganda [34]. The pro-rich distribution of hospitals and private providers may be similarly explained by the presence of financial barriers for the poor. In addition, these private facilities and hospitals are usually located in urban areas and are inaccessible to the poor.

Based on these results, for Uganda to achieve a more equitable distribution of health benefits, emphasis should be placed on improving lower level health service delivery. Likewise, the availability and accessibility of higher level health services should be guaranteed for all. Similarly, based on the progressivity of taxes and international experience, increasing general tax funding for health in Uganda and directing the resources at the lower levels of care where the poor are more likely to access them, will go a long way to addressing the mismatch between the need for health care and the distribution of health care in the country.

The major strength of this study is its ability to present a system-wide assessment of equity in both the financing and the benefits distribution of health services. Such a study is essential in order to inform Uganda’s strategy towards universal access to health care. However, this study has some limitations. The inability to distinguish between utilisation of inpatient and outpatient services based on the data set presents a major limitation in the assessment of the distribution of benefits. A further limitation is the use of the Ugandan national household survey data set in benefit incidence analysis particularly with regards to the difference in the nomenclature used in the survey and that used in the health system as has been noted by Orem et al. [10]. Likewise, the assessment of need within this context is still debatable. However, the paper follows those of previous studies in defining need [19,23,29,33,35]. If anything, the measure of need used in this paper may underestimate the actual distribution of need as reflected by population morbidity and mortality.In light of the above limitations, future studies should look at using data sets with improved measures of health care utilisation and more refined measures of need for assessing equity in the distribution of benefits.

## Conclusions

Recently, under the umbrella of universal health coverage, there is renewed interest in providing adequate health services to the population and ensuring that they do not experience financial ruin resulting from the use ofsuch services. This study provides evidence in the context of Uganda, a low-income country, about the extent to which health service benefits, measured in monetary terms, and the burden of health financing are distributed. Financing health care in Uganda is marginally progressive; placing lesser burdens on the poor relative to the rich. On the other hand, the distribution of health service benefits is generally to the advantage of the rich and the less needy. In order to ensure equity in the health system and to move the country toward universal coverage, there is a need for policy to focus on addressing the health needs of the poor and to continue ensuring that the burden of financing health services does not rest disproportionately on the poor.

## Endnotes

^a^This scale also assumes that male and female have similar calorie requirements.

^b^This adjustment is for only hospital based care as all visits to health units and clinics are assumed to be outpatient visits in line with previous studies in similar settings. See for example [29].

^c^Since out-of-pocket payments for these services obtained from this survey data are skewed, the median payment is used as the unit cost.
